# Prioritizing disease genes with an improved dual label propagation framework

**DOI:** 10.1186/s12859-018-2040-6

**Published:** 2018-02-08

**Authors:** Yaogong Zhang, Jiahui Liu, Xiaohu Liu, Xin Fan, Yuxiang Hong, Yuan Wang, YaLou Huang, MaoQiang Xie

**Affiliations:** 1 College of Software, Nankai University, TianJin, 300350 China; 2 School of Computer Science and Information Engineering, Tianjin University of Science and Technology, TianJin, 300222 China

**Keywords:** Label propagation, Gene prioritization, Heterogeneous network

## Abstract

**Background:**

Prioritizing disease genes is trying to identify potential disease causing genes for a given phenotype, which can be applied to reveal the inherited basis of human diseases and facilitate drug development. Our motivation is inspired by label propagation algorithm and the false positive protein-protein interactions that exist in the dataset. To the best of our knowledge, the false positive protein-protein interactions have not been considered before in disease gene prioritization. Label propagation has been successfully applied to prioritize disease causing genes in previous network-based methods. These network-based methods use basic label propagation, i.e. random walk, on networks to prioritize disease genes in different ways. However, all these methods can not deal with the situation in which plenty false positive protein-protein interactions exist in the dataset, because the PPI network is used as a fixed input in previous methods. This important characteristic of data source may cause a large deviation in results.

**Results:**

A novel network-based framework IDLP is proposed to prioritize candidate disease genes. IDLP effectively propagates labels throughout the PPI network and the phenotype similarity network. It avoids the method falling when few disease genes are known. Meanwhile, IDLP models the bias caused by false positive protein interactions and other potential factors by treating the PPI network matrix and the phenotype similarity matrix as the matrices to be learnt. By amending the noises in training matrices, it improves the performance results significantly. We conduct extensive experiments over OMIM datasets, and IDLP has demonstrated its effectiveness compared with eight state-of-the-art approaches. The robustness of IDLP is also validated by doing experiments with disturbed PPI network. Furthermore, We search the literatures to verify the predicted new genes got by IDLP are associated with the given diseases, the high prediction accuracy shows IDLP can be a powerful tool to help biologists discover new disease genes.

**Conclusions:**

IDLP model is an effective method for disease gene prioritization, particularly for querying phenotypes without known associated genes, which would be greatly helpful for identifying disease genes for less studied phenotypes.

**Availability:**

https://github.com/nkiip/IDLP

## Background

Disease gene prioritization aims to identify potential disease causing genes for a query phenotype. The accurate identification of corresponding disease genes is the first step toward a systematic understanding of the molecular mechanisms of a complex disease. Also, it is essential to know disease-related genes for diagnosis and drug development [6]. However, identifying disease-related genes is not an easy work, which is still one of the major challenges in the field of bioinformatics.

With the accumulation of studies on system biology, researches have shown genes that are physically or functionally close to each other tend to be involved in the same biological pathways and have similar effects on phenotypes [9, 22]. Based on such assumption, many network-based prioritization approaches have been developed to prioritize candidate genes [12, 13, 16, 26, 30, 31]. Early algorithms prioritize candidate genes based on their similarity to known disease genes [13, 26]. Though such type of methods perform well, they still have two limitations. The first limitation is caused by the fact that these methods only consider label propagation on homogeneous network (i.e. the PPI network). Thus, these methods easily fail when few disease-related genes are known. Later, methods that integrate heterogeneous networks have been proposed. By propagating label on both PPI network and phenotype similarity network [12, 16, 31], the prediction results have been boosted. Nevertheless, there is another limitation. As we know, high-throughput technologies have produced vast amounts of protein-protein interaction data. However, imprecise measuring technology brings a large number of false-positives in current available protein-protein interaction data [19, 20, 28]. Due to the alternating iterative learning approach adopted by previous methods [12, 16, 31], the PPI network can only be used as a fixed input, the false positive interactions between proteins in the PPI network will introduce a bias, and these noisy data are likely to result in less satisfying performance.

To tackle these challenges, we propose an Improved Dual Label Propagation (IDLP) method. Our motivation is inspired by label propagation [34] and the false positive protein-protein interactions in PPI network. Label propagation on homogeneous network and the associations between genes and phenotypes inspire us to construct the dual label propagation framework on heterogeneous network, and the false positive protein-protein interactions inspires us to regard the PPI network as a variable needed to be learnt rather than a fixed input. We construct a heterogeneous network by connecting the gene network and the phenotype similarity network with gene-phenotype associations. The basic label propagation (LP) [34] framework is extended from the homogeneous network to dual label propagation on the heterogeneous network. Query disease phenotypes and query disease genes are selected as seed nodes alternatively to propagate labels on the heterogeneous network. After that, an improved dual label propagation (IDLP) framework is proposed to reduce the bias introduced by false positive protein-protein interactions. The PPI network adjacent matrix is considered as a variable to be learnt under IDLP framework, its values are amended from noises by optimizing the loss function of IDLP. In case of overfitting to the training data, an additional regularization term is introduced to constrain the values in the PPI network matrix to be consistent with its initial values. The same regularization term is introduced to the phenotype similarity network as well. The objective matrices are optimized by minimizing the loss function. Furthermore, we propose an effective closed-form solution to improve calculation efficiency.

Our contribution can be summarized in the following two parts. 1) It’s the first time that the basic label propagation is extended from homogeneous networks to heterogeneous networks by directly modeling the loss function between labeled data and unlabeled data, through which it’s possible for us to take additional constraints into the loss function. On the contrary, alternating iteration strategy adopted by almost all previous works cannot deal with constraints efficiently. 2) It’s the first time that false positive PPIs have been taken into consideration, this bias regularization term greatly helps us to reduce the disturbance of data and improves the prediction accuracy in gene-phenotype prediction task.

## Methods

### Materials

We downloaded two versions (Aug-2015 version and Dec-2016 version) of human gene-phenotype associations from OMIM database [10]. The Aug-2015 version consists of 5117 associations between 4392 phenotypes and 3400 genes, and the Dec-2016 version contains 5465 associations between 4741 disease phenotypes and 3638 genes. The human protein-protein interaction (PPI) network was obtained from BioGRID [5] in August 2015. The PPI network contains 356,720 binary interactions between 19,511 genes. The disease phenotype network is an undirected graph with 8004 vertices representing OMIM disease phenotypes, the disease phenotype similarity between two phenotypes is calculated by text mining [25]. After filtering out isolated genes and disease phenotypes, we obtained 4,678/4,801 associations (Aug-2015/Dec-2016) between 4120 disease phenotypes and 3292 genes, corresponding PPI network and disease phenotype similarity network are extracted as well. More information about the data used in experiments is described in Table 1.

**Table 1:** Statistics of Data in Experiments

Statistics	Value
Number of genes	3292
Number of phenotypes	4120
Number of gene phenotype associations	4,678/4,801
(Aug-2015/Dec-2016)	
Average number of genes per phenotype	1.1354/1.1653
(Aug-2015/Dec-2016)	
Average number of phenotypes per gene	1.4210/1.4584
(Aug-2015/Dec-2016)	
Percentage of phenotypes that have only	91.87%/94.10%
one disease gene (Aug-2015/Dec-2016)	
Percentage of genes that have only one	66.22%/66.74%
interaction phenotype (Aug-2015/Dec-2016)	
Sparsity of the PPI matrix (Aug-2015)	99.74%

### Notations

Let *n* be the number of genes, *m* be the number of phenotypes, $\boldsymbol {W}_{1}\in \mathbb {R}^{n\times n}$ be the binary PPI network, and $\boldsymbol {W}_{2}\in \mathbb {R}^{m\times m}$ be the phenotype similarity network. ***W***_1_ and ***W***_2_ are used to construct normalized networks $\bar {\boldsymbol {S}}_{1}=\boldsymbol {D}_{1}^{-\frac {1}{2}}\boldsymbol {W}_{1}\boldsymbol {D}_{1}^{-\frac {1}{2}}$ and $\bar {\boldsymbol {S}}_{2}=\boldsymbol {D}_{2}^{-\frac {1}{2}}\boldsymbol {W}_{2}\boldsymbol {D}_{2}^{-\frac {1}{2}}$, and ***D***_*i*_ (i=1,2) is a diagonal matrix with the row-sum of corresponding ***W***_*i*_ (i=1,2) on the diagonal entries. The known gene-phenotype associations are represented by a binary matrix $\hat {\boldsymbol {Y}}_{(n \times m)}$ with 1 for entries of known associations and 0 otherwise. Let ***Y*** be the gene-phenotype associations matrix, ***S***_1_, ***S***_2_ be weighted PPI network and weighted phenotype similarity network, respectively. ***Y***, ***S***_1_, ***S***_2_ are the variables needed to be learnt. The notations used in the models are summarized in Table 2.

**Table 2:** Notations

NOTATION	DESCRIPTION
*n*	Number of genes
*m*	Number of phenotypes
***X***_*i*∙_	*i*-th row of matrix ***X***
***X***_∙*j*_	*j*-th column of matrix ***X***
$\boldsymbol {W}_{1}\in \mathbb {R}^{n\times n}$	Binary PPI network
$\boldsymbol {W}_{2}\in \mathbb {R}^{m\times m}$	Phenotype similarity network
$\bar {\boldsymbol {S}}_{1}\in \mathbb {R}^{n\times n}$	Normalized PPI network
	$\bar {\boldsymbol {S}}_{1}=\boldsymbol {D}_{1}^{-\frac {1}{2}}\boldsymbol {W}_{1}\boldsymbol {D}_{1}^{-\frac {1}{2}}$
$\bar {\boldsymbol {S}}_{2}\in \mathbb {R}^{m\times m}$	Normalized phenotype similarity network
	$\bar {\boldsymbol {S}}_{2}=\boldsymbol {D}_{2}^{-\frac {1}{2}}\boldsymbol {W}_{2}\boldsymbol {D}_{2}^{-\frac {1}{2}}$
$\hat {\boldsymbol {Y}}\in \mathbb {R}^{n\times m}$	Known binary gene-phenotype associations for training
$\boldsymbol {{Y}}\in \mathbb {R}^{n\times m}$	Gene-phenotype associations matrix to be learnt
${\boldsymbol {S}}_{1}\in \mathbb {R}^{n\times n}$	Weighted PPI network to be learnt
${\boldsymbol {S}}_{2}\in \mathbb {R}^{m\times m}$	Weighted phenotype similarity network to be learnt

### Problem definition

The goal of disease gene prioritization is trying to identify potential disease causing genes for a given phenotype. In our paper, we use variable ***Y*** as the gene-phenotype association matrix to be predicted. When finishing the optimization of the loss function, the genes with higher values in ***Y*** are predicted to be the potential disease causing genes for the given phenotype.

### Overall objective function

The overall objective function of IDLP is given in Eq. (1), and it includes *Ψ*_1_(***Y***,***S***_1_) and *Ψ*_2_(***Y***,***S***_2_), where *Ψ*_1_(***Y***,***S***_1_) is the objective function when label propagation is performed on the PPI network for all query phenotypes by considering the noises in the PPI network. *Ψ*_2_(***Y***,***S***_2_) is the objective function when label propagation is performed on the phenotype similarity network for all query genes by considering the noises in the phenotype similarity network.

1 $$  \begin{aligned} \boldsymbol{L}_{}(\boldsymbol{Y},\boldsymbol{S}_{1},\boldsymbol{S}_{2}) &= \Psi_{1}(\boldsymbol{Y},\boldsymbol{S}_{1})+\Psi_{2}(\boldsymbol{Y},\boldsymbol{S}_{2})\\ &=tr\left(\boldsymbol{Y}^{T}(\boldsymbol{I}-\boldsymbol{S}_{1})\boldsymbol{Y}\right)+tr\left(\boldsymbol{Y}(\boldsymbol{I}-\boldsymbol{S}_{2})\boldsymbol{Y}^{T}\right)\\ &\quad+ (\mu+ \zeta) ||\boldsymbol{Y}-\hat{\boldsymbol{Y}}||_{F}^{2}+\nu||\boldsymbol{S}_{1}-\bar{\boldsymbol{S}}_{1}||_{F}^{2}\\&\quad+\eta ||\boldsymbol{S}_{2}-\bar{\boldsymbol{S}}_{2}||_{F}^{2}. \end{aligned}  $$

where *μ*>0,*ζ*>0,*ν*>0,*η*>0. In the following subsections, we will explain how *Ψ*_1_(***Y***,***S***_1_) and *Ψ*_2_(***Y***,***S***_2_) are derived step by step. We also present a simple and descent solution of IDLP. Please note the algorithm of IDLP does not optimize the overall objective function directly, since variable ***Y*** can only be updated by gradient descend, and it’s very time consuming. In this paper, we optimize *Ψ*_1_(***Y***,***S***_1_) and *Ψ*_2_(***Y***,***S***_2_) alternatively to find a suboptimal solution, by which each variable can be updated with a closed-form solution.

### Dual label propagation on heterogeneous network

We introduce the conventional label propagation algorithm [34]. With a given query phenotype *p* and the PPI network ***W***_1_, the objective of label propagation is to learn an assignment score for each gene with the query phenotype *p*. The score shows how close each gene is to the query phenotype *p*. Let ${\hat {\boldsymbol {y}}}=\hat {\boldsymbol {Y}}_{\bullet p}$, i.e. the *p*-th column of the known association matrix $\hat {\boldsymbol {Y}}$. The non-zero elements in $\hat {\boldsymbol {Y}}$ are the initial labels on PPI network for query phenotype *p*. Let ***y***=***Y***_∙*p*_, i.e. the *p*-th column of the association matrix ***Y***. ***y*** is the label score vector of genes for query phenotype *p* needed to be learnt. Label propagation assumes that genes should be assigned with the similar label scores if they are connected in the PPI network, which leads to the following objective function,

2 $$ \begin{array}{ll} \Psi(\boldsymbol{y}) &=\sum\limits_{i,j}^{}(\boldsymbol{W}_{1})_{ij}\left(\frac{y_{i}}{\sqrt{\boldsymbol{D}_{ii}}} - \frac{y_{j}}{\sqrt{\boldsymbol{D}_{jj}}}\right)^{2} + \mu \sum\limits_{i}^{}(y_{i}-\hat{y}_{i})^{2}\\ &=\boldsymbol{y}^{T}(\boldsymbol{I}-\bar{\boldsymbol{S}_{1}})\boldsymbol{y} + \mu ||\boldsymbol{y}-\hat{\boldsymbol{y}}||^{2}, \end{array}  $$

where *y*_*i*_ is the *i*-th element of vector ***y***, $\hat {y}_{i}$ is the *i*-th element of vector $\hat {\boldsymbol {Y}}$. There are two terms in Eq. (2), *μ* (*μ*>0) is a parameter to balance the contributions of the two terms. The first term is the Laplacian graph constraint, which encourages consistent labeling in the PPI network. The second term is the regularization term to keep each node’s label value similar to its initial label value. Eq. (2) can be extended to predict associations for all the phenotypes as follows,

3 $$ \Psi_{1}(\boldsymbol{Y})=tr\left(\boldsymbol{Y}^{T}(\boldsymbol{I}-\bar{\boldsymbol{S}_{1}})\boldsymbol{Y}\right) + \mu ||\boldsymbol{Y}-\hat{\boldsymbol{Y}}||_{F}^{2}.  $$

On the other hand, with a given query gene *g* and phenotype similarity network ***W***_2_, the objective of label propagation is to assign a score for each phenotype with query gene *g*, the score shows how close each phenotype is to gene *g*. Phenotypes should be assigned with the similar labels if they have a high score in the phenotype similarity network for a given gene. Let $\hat {\boldsymbol {z}}=\hat {\boldsymbol {Y}}_{g \bullet }$, i.e. the *g*-th row of the known association matrix $\hat {\boldsymbol {Y}}$. The non-zero elements in $\hat {\boldsymbol {z}}$ is the initial labels on phenotype similarity network for query gene *g*. Let ***z***=***Y***_*g*∙_, i.e. the *g*-th row of the association matrix ***Y***. ***z*** is the label vector for query gene *g* needed to be learnt. Label propagation on phenotype similarity network for a given query gene *g* can be expressed as follows,

4 $$ \begin{array}{ll} \Psi(\boldsymbol{z}) &=\sum\limits_{i,j}^{}(\boldsymbol{W}_{2})_{ij}\left(\frac{z_{i}}{\sqrt{\boldsymbol{D}_{ii}}} - \frac{z_{j}}{\sqrt{\boldsymbol{D}_{jj}}}\right)^{2} + \zeta \sum\limits_{i}^{}(z_{i}-\hat{z}_{i})^{2}\\ &=\boldsymbol{z}(\boldsymbol{I}-\bar{\boldsymbol{S}_{2}})\boldsymbol{z}^{T} + \zeta ||\boldsymbol{z}-\hat{\boldsymbol{z}}||^{2}, \end{array}  $$

where *z*_*i*_ is the *i*-th element of vector ***z***, $\hat {z}_{i}$ is the *i*-th element of vector $\hat {\boldsymbol {z}}$. *ζ* (*ζ*>0) is a parameter to balance the contributions of the two terms in Eq. (4). Similar to the extension of Eqs. (2), (4) can be extended to predict associations for all the genes as follows,

5 $$ \Psi_{2}(\boldsymbol{Y})=tr\left(\boldsymbol{Y}(\boldsymbol{I}-\bar{\boldsymbol{S}_{2}})\boldsymbol{Y}^{T}\right) + \zeta ||\boldsymbol{Y}-\hat{\boldsymbol{Y}}||_{F}^{2}.  $$

### Improved dual label propagation on heterogeneous network

The false positive protein interactions in the PPI network indicate that $\bar {\boldsymbol {S}_{1}}$ contains noises. Therefore an intuitive idea is to introduce a variable ***S***_1_, trying to capture the real interaction relationship of genes. We replace the constant matrix $\bar {\boldsymbol {S}_{1}}$ with a variable matrix ***S***_1_ in Eq. (2), we can get the transformed Laplacian constraint term ***y***^*T*^(***I***−***S***_1_)***y***, then introduce the regularization term $\sum _{i,j}^{}((\boldsymbol {S}_{1})_{ij}-(\bar {\boldsymbol {S}}_{1})_{ij})^{2}$ to keep the interaction values similar to its initial values. The noises can be removed by optimizing these two components in terms of ***S***_1_. This leads to the following loss function for a given query phenotype *p*,

6 $$ {{} \begin{aligned} \begin{array}{ll} {}\Psi^{}(\boldsymbol{y},\boldsymbol{S}_{1}) &=\!\boldsymbol{y}^{T}(\boldsymbol{I}\,-\,\boldsymbol{S}_{1})\boldsymbol{y} \,+\, \mu ||\boldsymbol{y}\,-\,\hat{\boldsymbol{y}}||^{2} \,+\, \nu \sum\limits_{i,j}^{}((\boldsymbol{S}_{1})_{ij}-(\bar{\boldsymbol{S}}_{1})_{ij})^{2}\\ &=\boldsymbol{y}^{T}(\boldsymbol{I}-\boldsymbol{S}_{1})\boldsymbol{y} + \mu ||\boldsymbol{y}-\hat{\boldsymbol{y}}||^{2}+ \nu ||\boldsymbol{S}_{1}-\bar{\boldsymbol{S}}_{1}||_{F}^{2}, \end{array} \end{aligned}}  $$

Eq. (6) can be extended to predict associations with all the phenotypes as follows,

7 $$ {\begin{aligned} \Psi_{1}^{}(\boldsymbol{Y},\boldsymbol{S}_{1})&=tr\left(\boldsymbol{Y}^{T}(\boldsymbol{I}-\boldsymbol{S}_{1})\boldsymbol{Y}\right) \\&\quad+ \mu ||\boldsymbol{Y}-\hat{\boldsymbol{Y}}||_{F}^{2} + \nu ||\boldsymbol{S}_{1}-\bar{\boldsymbol{S}}_{1}||_{F}^{2}. \end{aligned}}  $$

To minimize the loss function in Eq. (7), an alternative iterative schema is adopted. It solves the problem with respect to one variable while fixing other variables. The loss function in Eq. (7) is not convex on ***Y*** and ***S***_1_ jointly, but it is convex on one variable with the other fixed.

In terms of Eq. (7), the closed form solutions of ***Y*** and ***S***_1_ can be expressed as,

8 $$ \begin{array}{ll} \boldsymbol{Y}^{*} &= \beta (\boldsymbol{I}-\alpha\boldsymbol{S}_{1})^{-1}\hat{\boldsymbol{Y}}\\ \alpha &= \frac{1}{1+\mu}, \ \ \beta = \frac{\mu}{1+\mu}\\ \boldsymbol{S}_{1}^{*} &= \bar{\boldsymbol{S}_{1}}+\gamma\boldsymbol{Y}\boldsymbol{Y}^{T},\ \ \gamma=\frac{1}{2\nu} \\ \end{array}  $$

**Fig. 1:**
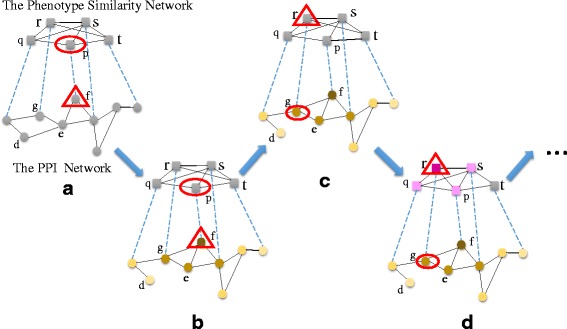
Illustration of the IDLP framework. Square nodes represent phenotypes, all pairwise phenotype similarity relationships make up the phenotype similarity network. Circular nodes represent genes, all pairwise gene interactions make up the PPI network. Nodes surrounded by oval are query phenotypes (or genes), Nodes surrounded by triangle are seed genes (or phenotypes). **a** For a query phenotype *p*, the corresponding related genes are selected as seed nodes. **b** By modeling the noises in the PPI network, the interactions between gene nodes have been changed. In order to better explain the situation, we consider two extreme cases here, i.e., edge deletion and edge addition. During the optimization of IDLP, the interaction between gene *g* and *f* has been added, the interaction between gene *d* and *e* has been removed. The changes of the PPI network result in a high score on gene *g*, because gene *g* directly receive score from seed gene *f*. What’s more, gene *d* no longer receives scores from gene *e*, which indirectly results in gene *d* receives more support from gene *e*. **c** For a query gene *g*, the corresponding related phenotypes are selected as seed nodes. **d** By modeling the noises in the phenotype network, the similarity scores between phenotypes have been changed. The edge addition between phenotype *r* and *p* and edge deletion between phenotype *r* and *t* result in a high score on phenotype *p*

After the label propagation on the PPI network with modeling the noises, the result is shown in Fig. 1b. Besides the values of ***Y***, the weight of each edge in the PPI network ***S***_1_ has been updated as well.

The phenotype similarity matrix $\bar {\boldsymbol {S}_{2}}$ can also be considered as inaccurate similarity relationships of phenotypes. Then we introduce a variable ***S***_2_, trying to capture the real relationships of phenotypes. At first, we replace the constant matrix $\bar {\boldsymbol {S}_{2}}$ with a variable matrix ***S***_2_ in Eq. (4), we can get the transformed Laplacian constraint term ***z***(***I***−***S***_2_)***z***^*T*^, then introduce the regularization term $\sum _{i,j}^{}((\boldsymbol {S}_{2})_{ij}-(\bar {\boldsymbol {S}}_{2})_{ij})^{2}$ to keep the predicted similarity values similar to its initial values. The noises can be removed by optimizing these two components in terms of ***S***_2_. This leads to the following loss function for a given query gene *g*,

9 $$  \begin{aligned} \Psi(\boldsymbol{z},\boldsymbol{S}_{2}) &=\!\boldsymbol{z}(\boldsymbol{I}-\boldsymbol{S}_{2})\boldsymbol{z}^{T} \,+\, \zeta ||\boldsymbol{z}\,-\,\hat{\boldsymbol{z}}||^{2} \,+\, \eta \sum\limits_{i,j}^{}\left((\boldsymbol{S}_{2})_{ij}\,-\,(\bar{\boldsymbol{S}}_{2})_{ij}\right)^{\!2}\\ &=\boldsymbol{z}(\boldsymbol{I}-\boldsymbol{S}_{2})\boldsymbol{z}^{T} + \zeta ||\boldsymbol{z}-\hat{\boldsymbol{z}}||^{2} + \eta ||\boldsymbol{S}_{2}-\bar{\boldsymbol{S}}_{2}||_{F}^{2}, \end{aligned}  $$

Eq. (9) can be extended to predict associations for all the genes as follows,

10 $$ {{} \begin{aligned} \Psi_{2}^{}(\boldsymbol{Y},\boldsymbol{S}_{2})\,=\,tr(\boldsymbol{Y}(\boldsymbol{I}\,-\,\boldsymbol{S}_{2})\boldsymbol{Y}^{T}) \,+\, \zeta ||\boldsymbol{Y}\,-\,\hat{\boldsymbol{Y}}||_{F}^{2} \,+\, \eta ||\boldsymbol{S}_{2}-\bar{\boldsymbol{S}}_{2}||_{F}^{2}. \end{aligned}}  $$

In terms of Eq. (10), the closed form solutions of ***Y*** and ***S***_2_ can be expressed as,

11 $$ \begin{array}{ll} \boldsymbol{Y}^{*} &= {\beta}^{'} \hat{\boldsymbol{xY}}(\boldsymbol{I}-{\alpha}^{'}\boldsymbol{S}_{2})^{-1}\\ \alpha^{'} &= \frac{1}{1+\zeta}, \ \ \beta^{'} = \frac{\zeta}{1+\zeta}\\ \boldsymbol{S}_{2}^{*} &= \bar{\boldsymbol{S}_{2}}+\gamma^{'}\boldsymbol{Y}^{T}\boldsymbol{Y},\ \ \gamma^{'}=\frac{1}{2\eta} \\ \end{array}  $$

Figure 1d shows the result after the label propagation on phenotype network by considering the noises in the phenotype similarity network. Besides the values of ***Y***, the phenotype similarity network ***S***_2_ has also been updated. The illustration of the IDLP is shown in Fig. 1. The algorithm details of IDLP are shown in Algorithm 1.



### Discussion about the Algorithm

Based on the Kurdyka-Lojasiewicz inequality [2] and the convexity of multi-variable objective function, when concentrating only on one of the variables at a time, our algorithm is convergent. Mostly it’s necessary to use the alternative iterative method, also known as “block coordinate descent” [18], to find the solution of multi-variable objective function. The objective function described in the manuscript is convex with concentrating exclusively on only changing one of the variables at a time, while the remaining variables are held fixed. This convex optimization problem satisfies the convergence condition for multi-variable objective function [32], and the Kurdyka-Lojasiewicz inequality [2] is used to prove the convergence.

There is one thing about inverse matrix we should notice. The computation of inverse matrix is a common step in optimization problems [7, 33]. In general, it’s time-consuming to compute inverse matrix with the adjugate-determinant method. In order to improve the efficiency of our algorithm IDLP, the inverse matrix is achieved by using Gaussian elimination. It is two times faster with Gaussian elimination in the experiments. It’s much more accurate and efficient with Gaussian elimination even when the matrix becomes very large.

Please be aware that the order of updating ***S***_1_ and ***S***_2_ can be exchanged, because the order does not change the convergence of the objective function. Besides the algorithm presented in the “Algorithm 1”, it’s also a feasible way to start the algorithm with the updates of ***S***_2_ and ***Y***, and then it comes with the updates ***S***_1_ and ***Y***. No matter which one comes first, they both result in reducing the objective function to the convergence.

### Theoretical analysis

#### BiRW is a special case of IDLP

BiRW [31] iteratively extends the phenotype path and the gene path by bi-random walk on both phenotype network and gene network to evaluate potential candidate associations. BiRW uses “Left Walk” and “Right Walk” alternatively to introduce additional steps on phenotype network and gene network. However, the loss function introduced by BiRW is rather misleading, which makes it impossible to get results by optimizing the loss function. The basic update rule for BiRW is:

Left walk on PPI network:

12 $$ \boldsymbol{Y}_{t} = \alpha\boldsymbol{S}_{1}\boldsymbol{Y}_{t-1}+(1-\alpha)\hat{\boldsymbol{Y}}.  $$

Right walk on phenotype network:

13 $$ \boldsymbol{Y}_{t} = \alpha\boldsymbol{Y}_{t-1}\boldsymbol{S}_{2} + (1-\alpha)\hat{\boldsymbol{Y}}.  $$

After sufficient left and right walks:

14 $$ \begin{array}{ll} \boldsymbol{Y}^{*}_{left} &= \beta(\boldsymbol{I}-\alpha\boldsymbol{S}_{1})^{-1}\hat{\boldsymbol{Y}}\\ \boldsymbol{Y}^{*}_{right} &= \beta\hat{\boldsymbol{Y}}(\boldsymbol{I}-\alpha\boldsymbol{S}_{2})^{-1}, \end{array}  $$

where *β*=1−*α*. Note that the solutions in (14) are exactly the same as in (8) and (11) when only the label propagation on heterogeneous network is considered without modeling the noise in data source. It shows that BiRW is a special case of IDLP. The final loss function for BiRW is as follows,

15 $$ \begin{array}{ll} \boldsymbol{L}_{BiRW}(\boldsymbol{Y}) &=tr\left(\boldsymbol{Y}(\boldsymbol{I}-\boldsymbol{S}_{2})\boldsymbol{Y}^{T}\right) + tr\left(\boldsymbol{Y}^{T}(\boldsymbol{I}-\boldsymbol{S}_{1})\boldsymbol{Y}\right)\\ &\quad+ \mu ||\boldsymbol{Y}-\hat{\boldsymbol{Y}}||_{F}^{2}+ \zeta ||\boldsymbol{Y}-\hat{\boldsymbol{Y}}||_{F}^{2}. \end{array}  $$

### Software package

A MATLAB software package is available through GitHub at https://github.com/nkiip/IDLP, containing all the source code used to run IDLP. The package allows the execution of cross-validation for parameter selection and model training with the selected optimal parameters to reproduce the results.

## Results

### Baselines

We compare our methods to both classic and the state-of-the-art network-based algorithms. We give a brief introduction to the baselines used in our experiments. CIPHER employs the regression model to quantify the concordance between the candidate gene and the query phenotype, then candidate genes are ranked by the concordance score [30]. RWR and DK (Diffusion Kernel) prioritize candidate genes by use of random walk from known genes for a given disease [13]. RWRH extends RWR algorithm to the heterogeneous network, it makes better use of the phenotypic data by using the query phenotypes and corresponding genes as seed nodes simultaneously [16]. PRINCE uses the known disease relationships to decide an initial set of genes that are associated with a query disease phenotype, then it performs label propagation on the PPI network to prioritize disease genes [26]. MINProp is based on a principled way to integrate three networks in an optimization framework and performs iterative label propagation on each individual subnetwork [12]. BiRW performs random walk on PPI network and phenotype similarity network alternatively to enrich genome-phenome association matrix, then prioritizes disease genes based on the enriched association matrix [31]. Besides the methods introduced above, two variants of IDLP are also introduced, i.e., IDLP-G and IDLP-P. In specific, IDLP-G assumes only the PPI network is noisy, where we set *η* to 0 in Eq. (10). IDLP-P assumes only the phenotype similarity network is noisy, where we set *ν* to 0 in Eq. (7).

### Experimental settings

IDLP has four parameters, i.e. *α*, *γ*, $\alpha ^{'}\phantom {\dot {i}\!}$, $\gamma ^{'}\phantom {\dot {i}\!}$. Since the constraint *α*+*β*=1 and $\alpha ^{'}+\beta ^{'} = 1\phantom {\dot {i}\!}$, the value of *β* and $\beta ^{'}\phantom {\dot {i}\!}$ are fixed when *α* and $\alpha ^{'}\phantom {\dot {i}\!}$ are chosen. For the data of training in cross-validation, we select parameter values by using a usual manner of (5-fold) cross-validation: only a part (four folds) of the training dataset is used for getting model results of IDLP, meanwhile the rest (one fold) for validation, this is done five times with each fold as validation set in turns. The average results of the five folds are used for choosing best parameters. In parameter selection, we consider all combinations of the following values: {0.0001, 0.001, 0.01, 0.1, 1} for *α* and $\phantom {\dot {i}\!}\alpha ^{'}$, {1, 10, 100, 1000, 10000} for *γ* and $\gamma ^{'}\phantom {\dot {i}\!}$.

We implement all the baselines according to the descriptions in their papers. CIPHER doesn’t have any parameters to tune, so it is applied to the test set directly. For RWR, DK, and PRINCE, they are network-based methods only walk on gene interaction network, the parameter *α* is chosen from {0.1, 0.3, 0.5, 0.7, 0.9} by 5-fold cross-validation. For RWRH, MINProp and BiRW, they perform a random walk on a heterogeneous network of gene interactions and human diseases (i.e. OMIM phenotypes similarity network). We use the average version of BiRW which is shown to be the best among the three versions of BiRW proposed by Xie [31], and the left and right walk step are set to 4 as suggested by Xie. There is one parameter in BiRW, which is chosen from {0.1, 0.3, 0.5, 0.7, 0.9} by cross-validation. There are two parameters in MINProp, which are chosen from {0.1, 0.3, 0.5, 0.7, 0.9} by grid through cross-validation.There are three parameters in RWRH, which are all chosen from {0.1, 0.3, 0.5, 0.7, 0.9} by grid search.

### Evaluation

We evaluated the ranks of the tested genes with two metrics: (i) we calculated the area under the curve (AUC) [8, 11] for each method. “AUC” refers to the area under a Receiver Operating Characteristic (ROC) Curve, and the result is a plot of true positive rate against false positive rate. (ii) we calculated the average precision and recall on test set at top-k positions (k=20, 50, 100). The two metrics are complimentary: the AUC evaluates the entire rank of genes, while the top-k precision and recall emphasize the top-ranked genes.

Since the accuracy of top-ranked genes is more important than that of the lower ranked genes, we highlight a set of false positive cutoffs for the ROC curves and compare the corresponding average AUCs between methods. The higher the AUC score, the better the performance.

Conventional cross-validation evaluation strategy, such as leave-one-out cross-validation strategy, does not necessarily reflect the property of novel gene-phenotype associations prediction. To address such cases, we adopt the strategy that has been utilized by [21, 23, 31], i.e. two versions of data are used in the experiments, the Aug-2015 version data are used as validation set to train the model, the newly added data accumulated between Aug-2015 and Dec-2016 are used as test set to measure the performance of the model. In the experiment, we split the known gene-disease associations of Aug-2015 version data into five folds. After doing 5 folds cross-validation, the average results of the five folds are used for selecting parameters for each method. Then, the methods are applied to predict the associations in an independent set of associations added into OMIM between Aug-2015 and Dec-2016.

### Performance evaluation

To quantitatively evaluate IDLP and other baseline methods, i.e. CIPHER, RWR, DK, RWRH, MINProp, BiRW, and PRINCE, these algorithms are applied to predict the disease genes for each phenotype.

The performance of IDLP and baseline methods on test set and cross-validation set are shown in Table 3. We have conducted Student’s t-test [3] with *p*<0.05 on the results of IDLP and other baselines on test set. If IDLP outperforms one baseline significantly under AUC metric, we put a “*” behind the performance value in Table 3. The performance results on cross-validation are used for choosing parameters for each method. RWRH gets the best results on cross-validation set. However, the performance of RWRH on test set dramatically falls compared with that of IDLP. RWRH heavily depends on the completeness and correctness of PPI network and phenotype similarity network, which brings the serious overfitting. It can be seen that IDLP achieves the best performance under AUC20 and AUC50 on test set, which means the proposed IDLP can predict newly discovered gene-phenotype associations well. By introducing the dual label propagation framework and modeling the bias in the PPI network and phenotype similarity network into the framework, it successfully utilizes the information in the heterogeneous network and overcomes the interference of the noises in data source. This demonstrates the advantage of IDLP over other baselines.

**Table 3:** Average AUCs scores of gene prioritization on test set and validation set

	Performance on test set			Performance on validation set		
	AUC20	AUC50	AUC100	AUC20	AUC50	AUC100
CIPHER_SP	0.0029^*^	0.0046^*^	0.0066^*^	0	0	0
CIPHER_DN	0.0015^*^	0.0027^*^	0.0042^*^	0	0	0
RWR	0.0075^*^	0.0178^*^	0.0283^*^	0.0233	0.0358	0.0475
DK	0.0192^*^	0.0255^*^	0.0294^*^	0.0211	0.0306	0.0399
RWRH	0.0916^*^	0.1250^*^	0.1664^*^	0.2009	0.2724	0.3288
MINProp	0.0771^*^	0.1266^*^	0.1799^*^	0.1963	0.2625	0.3104
BiRW	0.0421^*^	0.0780^*^	0.1142^*^	0.1544	0.2180	0.26672
PRINCE	0.1117	0.1468	0.2088	0.1433	0.2137	0.2715
IDLP-G	0.0040^*^	0.0076^*^	0.0166^*^	0.0189	0.0348	0.0519
IDLP-P	0.1051^*^	0.1457	0.1897	0.2003	0.2592	0.3010
IDLP	0.1123	0.1492	0.1909	0.2004	0.2572	0.2990

We compared AUCs when the number of false positive genes are up to 20, 50, 100

^*^indicates IDLP significantly outperforms the baseline with *p*<0.05 using Student t-test

It can be observed that IDLP-P has a distinct advantage over IDLP-G in terms of AUC values on test set, which demonstrates the noises in the phenotype similarity network are serious, whether modeling the noises in the phenotype similarity network would greatly affect the results. We can also observe that IDLP-G performs worse than most of the baselines, which demonstrates that only modeling the noises in the PPI network will bring more noises to the model. The phenotype similarity network is constructed by calculating the similarity scores between phenotypes through text mining [25]. The calculation of the similarity scores depends on the terms of the descriptions of phenotypes, term frequencies, sentence expressions, etc. The integrity and the accuracy of the descriptions of phenotypes can greatly affect the similarity scores between phenotypes, hence the similarity scores are subjective and the phenotype similarity contains much noises. As for the PPI network, much of the data in it are collected from in vitro experiments. Thought imprecise measurement introduces false positives, there are still lots of true interactions between proteins. The data in the PPI network are more objective and contain less noises. Another reason that causes the difference between IDLP-G and IDLP-P is that the PPI network is a sparse network, and the phenotype similarity network is a dense network. The sparse network is more sensitive to changes in the network. The results comparison between IDLP and its two variants demonstrate that modeling the noises on both PPI network and phenotype similarity network is better than modeling the noises only on PPI network or phenotype similarity network. It indicates modeling the noises on both networks has a mutual enhancement to the results. Based on this fact, we will focus on IDLP and ignore its two variants in the following discussion.

**Fig. 2:**
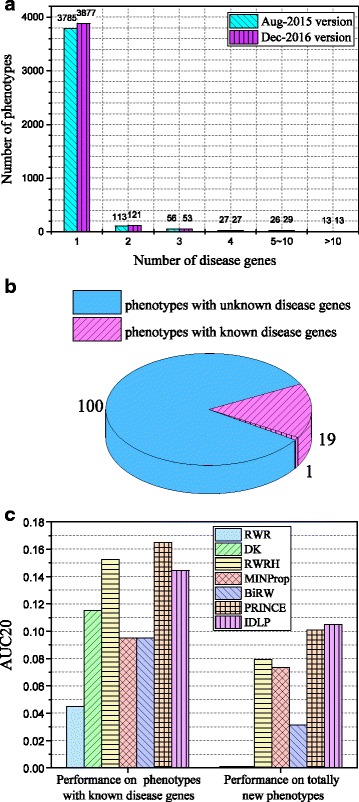
Data Analysis. **a** The phenotype distribution based on the genes it associates with. **b** The distribution of newly added phenotypes based on whether they have known disease causing gene(s). **c** The AUC20 scores of different methods in two situations: 1. phenotypes with known disease genes are used as queries (left); 2. phenotypes with unknown disease genes are used as queries (right)

In order to understand IDLP further, we give an analysis of the constitution of the data. Figure 2a shows the phenotype distribution of the two versions according to the disease genes they associate with. More specifically, there are 3785 phenotypes associated with one disease gene in Aug-2015 version data, the number of phenotypes increases to 3877 in Dec-2016 version data; the numbers of phenotypes which have been found with more than one disease genes change slightly. There are 123 newly added gene-phenotype associations. More specifically, as shown in Fig. 2b, 100 phenotypes are newly added to Dec-2016 version data, which means there are 100 phenotypes with unknown disease genes in Aug-2015 version data. The remaining 23 associations can be divided into 2 categories, 19 phenotypes with known disease genes being added with one more disease gene and 1 phenotype with known disease genes being added with 4 new disease genes. From Fig. 2a and b, we know the phenotypes involved in newly added gene-phenotype associations between Aug-2015 version and Dec-2016 version are mostly phenotypes with unknown disease genes in Aug-2015 version. Here we define these phenotypes without any known disease genes as *singleton* phenotypes. Since the number of singleton phenotypes accounts for a large percentage, it is important and necessary to explore the performance on singleton phenotypes.

Figure 2c shows the results when different associations are used as test set. The left histogram in Fig. 2c shows the performance when 23 associations with none singleton phenotypes are used as test set. The right histogram in Fig. 2c shows the performance when 100 associations with only singleton phenotypes are used as test set. Because the results of CIPHER_SP and CIPHER_DN are too small in the histogram, we ignore them in this discussion. Comparing these two histograms in Fig. 2c, we can observe that predictions on phenotype queries that have known disease genes are more precise than phenotype queries that have non disease genes for each method. It is consistent with the intuition that enriched phenotypes (i.e. phenotypes with at least one known disease gene) are easier to find disease genes. RWRH, PRINCE, and IDLP have relatively high AUC20 scores on enriched phenotype queries. On the contrary, it’s hard to identify disease genes for singleton phenotypes, because no known disease genes are discovered for these singleton phenotypes. That’s why RWR and DK decrease to zero. Meanwhile, IDLP achieves best at this situation, which demonstrates IDLP’s effectiveness on singleton phenotypes.

### Noises discussion

Generally, there is no prior information on how the noises are in the data sources. When dealing with unknown imbalanced noises in the networks, a good algorithm can automatically choose proper penalty values on the noises. The proper penalty values are determined by the noise situations in the two networks, which means heavy noises correspond to big penalty value and small noises correspond to small penalty value. In IDLP, the algorithm is adaptive to imbalanced noises, and it can choose proper hyper parameters automatically by grid search of parameters on validation set.

Please note that IDLP may not work under some situations. IDLP may fail when data contain little noises. IDLP is designed for dealing with noise data, however unnecessary learning of variables from noises would deviate the model from clean data. That probably causes performance decline of the algorithm on test set. To avoid the failure, we’d better acquire some basic information about the data noises and decide whether to model the noises before applying IDLP.

### Top-k precision and recall evaluation

**Fig. 3:**
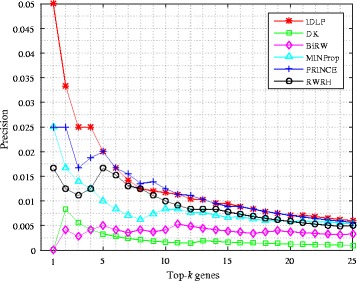
Average precision on all query diseases of test set at each top-k position

**Fig. 4:**
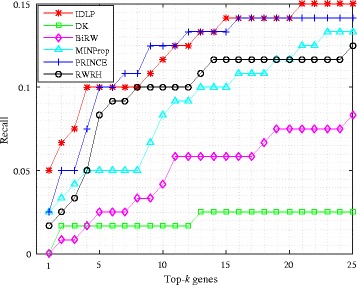
Average recall on all query diseases of test set at each top-k position

We also evaluate IDLP and baseline methods by using precision and recall measurement. Calculating precision and recall at each top-k position tells a more strict and detailed comparison between different methods. Precision measures the fraction of true positives (genes) recovered in the top-k predictions for a phenotype. Recall is the ratio of true positives recovered in the top-k predictions for a phenotype to the total number of true positives in the test set. The plot of top-k precision and recall rates for different values of top k positions ranging 1≤k≤25 is presented in Figs. 3 and 4 respectively. The value at a given k is averaged over all the phenotypes. Note that IDLP outperforms other baselines, especially when 1≤k≤5. For example, in Fig. 3 the precision at top 1 position of IDLP is 0.05, it’s twice as much as the second best precision 0.025 of PRINCE and MINProp. In Fig. 4, the recall at top 1 position of IDLP is 0.05, and that’s also twice as much as the second best recall 0.025 of PRINCE and MINProp. IDLP outperforms on both precision and recall when k is less than 5, especially at top 1 position. The superiority of IDLP at top-k precision and recall demonstrates the effectiveness of modeling the bias and denoising through dual label propagation framework.

## Discussion

### Sensitivity of parameter *α* and *γ*

**Fig. 5:**
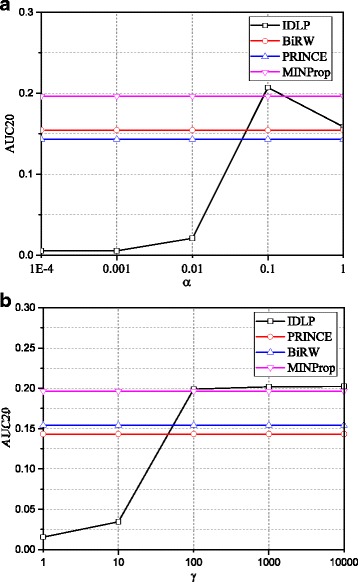
Effects of parameters on the performance of IDLP (**a**) Performance of AUC20 w.r.t *α*. **b** Performance of AUC20 w.r.t *γ*

Figure 5 shows the effects of the parameters. AUC20 is used as a measurement of performance. For IDLP, we fix *γ* = 1000 when varying *α* and fix *α* = 0.1 when varying *γ*. The performance of other methods is also presented for reference. We observe that IDLP is not sensitive when *γ* becomes large. Large *γ* will raise effect of ***YY***^*T*^ and reduce the effect of $\bar {\boldsymbol {S}_{1}}$ on updating ***S***_1_, when *γ* becomes large enough, the effect of $\bar {\boldsymbol {S}_{1}}$ disappears. In our experiments, we set *α* = $\alpha ^{'}\phantom {\dot {i}\!}$ = 0.1 and *γ* = $\gamma ^{'}\phantom {\dot {i}\!}$ = 1000 for IDLP.

### Robustness evaluation of IDLP

**Fig. 6:**
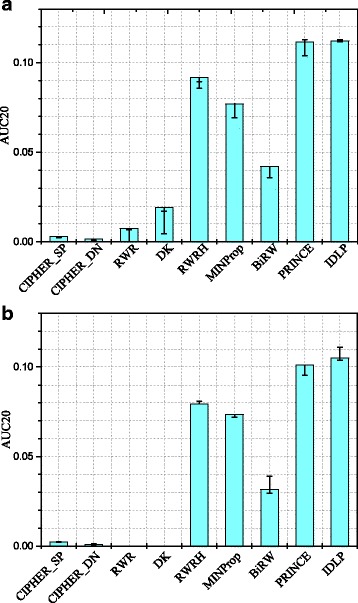
Robustness of IDLP. Four disturbed PPI networks are applied into each algorithm: 1. randomly delete 10% PPI data; 2. randomly delete 10% PPI data and add 10% PPI data; 3. randomly delete 20% PPI data; 4. randomly delete 20% PPI data and add 20% PPI data. The best and the worse performance of these four situations are drawn as error bar on the histogram. **a** It shows the results when all diseases are chosen as test set. **b** It shows the results when totally new diseases are chosen as test set

We check the AUC20 performance result for each method under four disturbed PPI networks: 1) randomly delete 10% PPI data; 2) randomly delete 10% PPI data and add 10% PPI data; 3) randomly delete 20% PPI data; 4) randomly delete 20% PPI data and randomly add 20% PPI data. The best and the worst performance of these four situations are drawn as error bars on the histogram. Figure 6a shows the result when choosing all disease phenotypes as test set, and we can see that IDLP has a greatly stable performance under all kinds of disturbance. Figure 6b shows the result when total new disease phenotypes are chosen as test set. The advantage has become more obvious when we only consider the total new phenotypes (i.e. singleton phenotypes defined above) as test set. From the results in Fig. 6, we can conclude that IDLP has a good robustness.

The robustness comes from the design of the loss function of IDLP. More specifically, the update mechanism determines the robustness of IDLP. Let us go over the first two steps of Algorithm 1. At first, ***S***_1_ is updated by $\boldsymbol {S}_{1}^{} \leftarrow \bar {\boldsymbol {S}_{1}}+\gamma \boldsymbol {Y}\boldsymbol {Y}^{T}$, then the gene-phenotype associations matrix ***Y*** is updated by $\boldsymbol {Y}^{} \leftarrow \beta (\boldsymbol {I}-\alpha \boldsymbol {S}_{1})^{-1}\hat {\boldsymbol {Y}}$. After sufficient iterative update, *γ****YY***^*T*^ has much influence on $\boldsymbol {S}_{1}^{}$ and the influence is even stranger when *γ* becomes a large value.

### Predicting new genes for Parkinson’s disease

Predictions of new genes for specific diseases are examined to check the prediction accuracy of IDLP. In the data we obtained, there are 30 genes known to be associated with Parkinson’s Disease (PD) on OMIM till December 2016. Apart from the known 30 disease genes for Parkinson’s Disease in OMIM data, other top 10 predicted genes are supposed to be most closely associated with PD according to the scores got from our proposed IDLP. We searched the literatures to support our predictions, the results are showed in Table 4. 8 (80%) of the top 10 genes have supporting evidence giving a prediction precision of 80% for this particular disease.

**Table 4:** Predicted top 10 new genes for Parkinson’s disease by IDLP

Gene	Score	Evidence of Support
DNAJC13	0.7016	DNAJC13 mutations in Parkinson disease [27].
CYP2D6	0.5796	CYP2D6 phenotypes and Parkinson’s disease risk: a meta-analysis [17].
DRD4	0.5667	Lack of allelic association of dopamine D4 receptor gene polymorphisms with Parkinson’s disease in a Chinese Population [29].
RAB39B	0.5421	Loss-of-function mutations in RAB39B are associated with typical early-onset Parkinson disease [15].
TRPM7	0.3101	TRPM7 and its role in neurodegenerative diseases [24].
SNCB	0.2342	Beta-synuclein gene variants and Parkinson’s disease: a preliminary case-control study [4].
DCTN1	0.1791	A Novel DCTN1 mutation with late-onset parkinsonism and frontotemporal atrophy [1].
ATP6AP2	0.1562	Altered splicing of ATP6AP2 causes X-linked parkinsonism with spasticity (XPDS) [14].
WDR45	0.1415	-
PSEN2	0.1401	-

The 10 genes listed in Table 4 have not been recorded in OMIM dataset. However, according to the calculation results by IDLP, they are highly PD related candidate genes. We search the literatures and try to find the connections between these genes and Parkinson’s Disease. Specifically, Vilarino discovered that idiopathic Parkinson’s disease subtle deficits in endosomal receptor-sorting/recycling are highlighted by the discovery of pathogenic mutations DNAJC13 [27]. Lu demonstrated that the poor metabolizer phenotype of CYP2D6 confers a significant genetic susceptibility to Parkinson’s disease in Caucasians [17]. Wan conducted experiments to test the hypothesis that the DRD4 polymorphism is associated with the susceptibility to Parkinson’s disease [29]. Lesage reported an additional affected man with typical Parkinson’s disease and mild mental retardation harboring a new truncating mutation in RAB39B [15]. Sun found the discrepancy in TRPM7 channel function and expression leads to Parkinson’s disease [24]. Laura’s study suggested that the SNCB locus might modify the age at onset of PD [4]. Araki found DCTN1 mutations may contribute to disparate neurodegenerative diagnoses, including familial motor neuron disease, parkinsonism, and frontotemporal atrophy [1]. Korvatska reported that X-linked parkinsonism with spasticity (XPDS) presents either as typical adult onset Parkinson’s disease or earlier onset spasticity followed by parkinsonism [14]. We briefly list all the top 10 predicted genes, prediction scores by IDLP and literatures evidence for Parkinson’s disease in Table 4.

## Conclusions

We propose an Improved Dual Label Propagation (IDLP) algorithm, which is based on optimizing the regularization framework, rather than alternating iteration used by previous works, to globally prioritize disease genes for all phenotypes. IDLP performs label propagation on the protein-protein interaction (PPI) network and the phenotype similarity network alternatively. Meanwhile, it models the noise disturbance of the false positive PPIs in the data source to get a better result. By amending the noise in training matrices, it improves the performance results significantly. We also give a closed-form solution, which makes the algorithm more efficient. In our experiments, we find that IDLP has an outstanding performance for ranking top genes and a good robustness to deal with the noise in PPI network, which makes IDLP a better gene prioritization tool for biologists.
